# Rats and humans differ in processing collinear visual features

**DOI:** 10.3389/fncir.2013.00197

**Published:** 2013-12-13

**Authors:** Philip M. Meier, Pamela Reinagel

**Affiliations:** ^1^Department of Neurosciences, Division of Medicine, University of California at San DiegoLa Jolla, CA, USA; ^2^Section of Neurobiology, Division of Biology, University of California at San DiegoLa Jolla, CA, USA

**Keywords:** rodent, collinearity, flanker task, visual perception, contrast, attention, psychophysics, cortical computation

## Abstract

Behavioral studies in humans and rats demonstrate that visual detection of a target stimulus is sensitive to surrounding spatial patterns. In both species, the detection of an oriented visual target is affected when the surrounding region contains flanking stimuli that are collinear to the target. In many studies, collinear flankers have been shown to improve performance in humans, both absolutely (compared to performance with no flankers) and relative to non-collinear flankers. More recently, collinear flankers have been shown to impair performance in rats both absolutely and relative to non-collinear flankers. However, these observations spanned different experimental paradigms. Past studies in humans have shown that the magnitude and even sign of flanker effects can depend critically on the details of stimulus and task design. Therefore either task differences or species could explain the opposite findings. Here we provide a direct comparison of behavioral data between species and show that these differences persist – collinear flankers improve performance in humans, and impair performance in rats – in spite of controls that match stimuli, experimental paradigm, and learning procedure. There is evidence that the contrasts of the target and the flankers could affect whether surround processing is suppressive or facilitatory. In a second experiment, we explored a range of contrast conditions in the rat, to determine if contrast could explain the lack of collinear facilitation. Using different pairs of target and flanker contrast, the rat’s collinear impairment was confirmed to be robust across a range of contrast conditions. We conclude that processing of collinear features is indeed different between rats and humans. We speculate that the observed difference between rat and human is caused by the combined impact of differences in the statistics in natural retinal images, the representational capacity of neurons in visual cortex, and attention.

## INTRODUCTION

Specialized interaction of nearby collinear features is thought to play an important role in contour integration and figure/ground segregation of scenes. In natural images, collinear features enjoy prominent statistical correlations across spatial regions and feature types. It has been suggested (Barlow, 1961; Olshausen and Field, 1996; Simoncelli and Olshausen, 2001; Coen-Cagli et al., 2012) that neurons learn to represent the world by exploiting the joint statistics between their inputs. Thus cortical computation may latch on to events induced by collinear stimuli, and appropriately enhance or suppress them. But what is appropriate? Are collinear features redundant, and should be suppressed in order to optimize the channel capacity of the neural code? Or are collinear features highly informative about scenes, and thus should be emphasized as salient features for subsequent processing? We argue that a good way to begin understanding the cortical code is to examine the neural and behavioral responses to stimuli with features that are correlated in the natural world, but are made independent in the course of the study. In this paper, we directly compare the behavioral responses of humans and rats detecting a visual target surrounded by collinear flankers.

Rodents are increasingly used as a model system for the study of cortex, including the visual system (Bussey et al., 2001; Niell and Stryker, 2008; Andermann et al., 2011; Bonin et al., 2011; Huberman and Niell, 2011; Meier et al., 2011; Reid, 2012; Alemi-Neissi et al., 2013; Haider et al., 2013). Many aspects of visual processing are conserved in the thalamo-cortical visual pathway of mammals, including center-surround antagonism, light adaptation, contrast adaptation, orientation tuning, spatial bandpass filtering, and phase selectivity. Yet, there are also differences between primates and rodents in the organization early visual processing (van den Bergh et al., 2010). These include differences in connectivity across layers of V1 (Zarrinpar and Callaway, 2006), as well as differences in organizational principles like orientation tuning maps (Ohki et al., 2005). When we learn about the function of rodent visual cortex, will it generalize to human vision? Mammals likely share many common computational goals in early vision, and achieve similar algorithmic solutions with the same biological components. In other respects, surely divergence or specialization will result in differences between species. Using multiple species to elucidate mechanisms of mammalian vision, it will be important to determine both the similarities and differences at each level of description.

A recent study in rodent behavior demonstrated perceptually guided behaviors in rats that are specific to collinear stimuli (Meier et al., 2011). All patterns of flanking stimuli (“flankers”) impair rats’ ability to detect a target stimulus. Collinear flankers impair their detection even more. This finding stands in contrast to previous reports of human psychophysics in which collinear flankers improve a human subject’s capacity to detect a central visual target (Polat and Sagi, 2007; Chen and Tyler, 2008). It was possible, however, that these differences between previous studies could be attributed to differences in stimuli, experimental paradigm, or learning procedure. For example, many of the experiments in the human literature do not vary the orientation of the target on each trial, such that feature-based attention could contribute to the observed effects. Before this study, there were no experiments on human visual detection with collinear flankers that controlled for the subject’s expectation of the orientation of the target feature.

Here we present a new study of both human perception and rodent behavior in which the parameters and experimental conditions were matched. For rats, we extend our previous finding that collinear flankers impair detection to a broader range of contrast conditions. For humans, we replicate the previous finding that collinear flankers improve human’s ability to detect visual targets, extending this result to a new task variant that includes controls which were lacking in past human studies. Together these findings constitute the first direct comparison that demonstrates that the perceptual mechanisms involved in processing collinear features differ between the species. Importantly, it is not simply that rats lack pattern-specific processing (sensitivity to higher order configurations or feature conjunctions). Both humans and rats demonstrate a perceptual sensitivity particular to collinear stimuli, but between the species, the sign of the effect is reversed.

## MATERIALS AND METHODS

**FIGURE 1:**
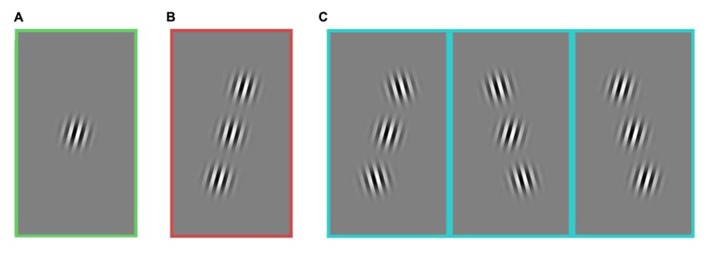
**Stimuli composed of a central target and two flanking stimuli.** All images include a target in the figure, but on 50% of trials, the central target was absent. Three stimulus groups used for the analysis of the first experiment: **(A)** no flanker, **(B)** collinear, and **(C)** non-collinear. The three kinds of non-collinear stimuli were grouped together. All stimulus categories **(A–C)** included horizontally reflected versions of the stimuli (not shown here). In this experiment, the contrast of every element was held constant. Rats and humans performed the same experiment. The task was to detect if the central target was present or absent.

**FIGURE 2:**
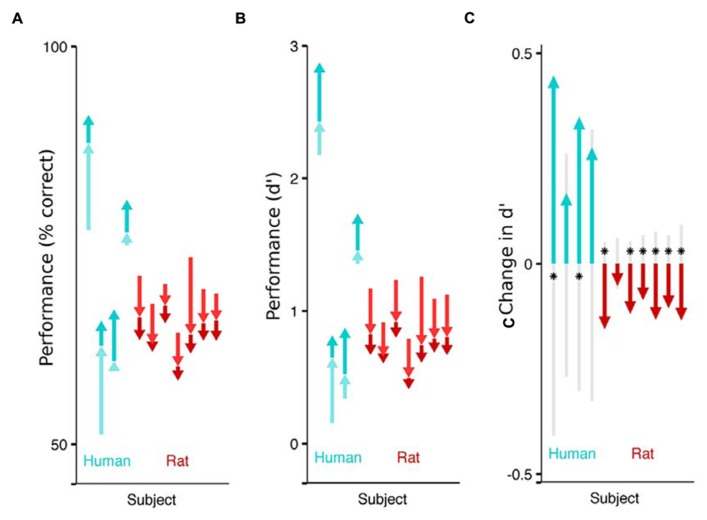
**Impact of flanking stimuli on the performance of humans and rats detecting a faint visual target.** Each arrow indicates the difference in fraction of correct trails across stimulus categories, for a single subject (cyan for humans, red for rats). The pale arrow denotes the impact of non-collinear flankers with respect to no flankers. The darker arrow indicates the additional impact of collinear flankers, above and beyond the effect of non-collinear flankers. The sum of the two arrows captures the difference in performance between detecting targets without any flankers, and detecting targets in the presence of collinear flankers. The absolute performance on each of the three stimulus conditions (collinear, non-collinear, no flank) is captured by the tip and the base of the arrows. **(A)** Effect of flankers on performance (% correct). Humans performed better on trials with collinear flanking stimuli (upwards arrows) and rats performed worse on the trials with collinear flanking stimuli (downwards arrows). **(B)** Effect of flankers on sensitivity (*d*’). **(C)** Each subject’s sensitivity on the collinear trials minus their sensitivity on the non-collinear trials. The gray shaded region indicates chance differences within the range spanned by 95% of 10,000 random permutations of the subject’s response with respect to the stimulus. If performance for a given subject is significant beyond this chance range, it is marked with an asterisk.

**FIGURE 3:**
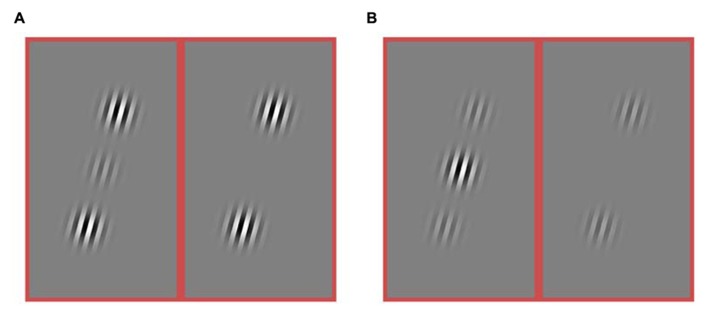
**Stimuli from an experiment in which both the target and flanker contrasts were varied.** The contrast of the target varied ([0.25, 0.5, 0.75, 1.0]). The contrast of the flankers was varied independently ([0, 0.25, 0.5, 0.75, 1.0]). **(A)** Example of a stimulus condition with high contrast flankers (1.0) and a low contrast target (0.25). The target is present in the left sub-panel and absent from the right sub-panel. **(B)** Example of a stimulus with a high contrast target (1.0) and low contrast flankers (0.25). Again, the left and right sub-panels differ by the presence vs. absence of the target. Only collinear flankers were used in this experiment.

**FIGURE 4:**
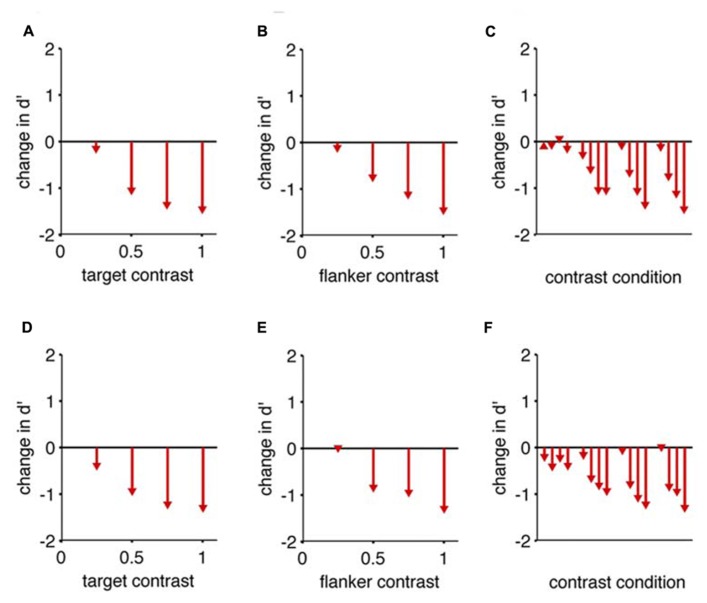
**Collinear flankers impair rats regardless of contrast condition.** Performance of two rats in which the target contrast and the flanker contrasts were independently varied. In all cases, we measured *d*’ for detection of a target with collinear flankers, and subtracted the *d*’ we measured for the same target contrast with no flankers (reference condition). This difference (change) in *d*’ is indicated by arrowheads in the bar graphs. The base of each arrow is zero by definition because the flanker condition is the reference condition. **(A)** The reduction in detection performance caused by full contrast flankers, at four different target contrasts. **(B)** The reduction in detection performance caused by four different flanker contrasts, for a full contrast target. **(C)** All possible combinations of target contrast and flanker contrast impaired the rat’s performance. The reference condition for each comparison has no flanker present, and an equivalent target contrast. Eight of the 16 comparisons are identical to panels **(A,B)**. Panels **(A–C)** show data from one subject; **(D–F)** show equivalent data from the second subject.

In the first experiment, the spatial patterns of stimuli were varied, while the contrast was held constant (**Figures 1** and **2**). In the second experiment, spatial patterns were held constant and the contrast of stimuli was varied (**Figures 3** and **4**).

### EXPERIMENT 1

Both humans and rats performed the same detection task. Subjects performed one of two symmetric actions to indicate either that the target grating was present in the center of the screen, or that it was absent. If the two flanking stimuli were present, they were located on opposite sides of the target, with a diagonal offset (**Figure 1**).

Compared to most experiments that explore the influence of collinear flankers in human perception, this experiment differs in three ways. Instead of receiving instructions, humans learned from trial and error that it was a detection task. Instead of viewing sine-wave gratings, humans viewed square-wave gratings. And instead of performing the detection task on all of the collinear trials in a row, humans viewed collinear trials that were randomly interleaved with other non-collinear patterns. Within the controlled comparisons of this study, all three of these traits were consistent for both species.

First, both humans and rats learned to perform correct trials by trial and error. Rats licked one of three ports; humans pressed one of three buttons. The central port/button initiated a new trial. The ports/buttons on the left and right side indicated either “target present” or “target absent”; these meanings were randomly assigned for each subject. Rats were motivated to collect water rewards, and humans were instructed to seek the incidences of positive tones that were audible after completing a trial correctly. Second, both humans and rats viewed target stimuli that were oriented gratings with a square wave pattern. Both viewed stimuli frontally, such that binocular vision could be used. Both viewed stimuli that were 32 pixels per cycle on the screen, but rats viewed from a distance 10 cm, resulting in a target 0.15 cpd in a Gaussian envelope with a STD of 10 degrees, and humans viewed from a distance of 2.15 m, resulting in a target 3.3 cpd in a Gaussian envelope with a STD of 0.45 degrees. Note that in both species, the Gaussian envelope of the grating, in degrees, maintained a fixed proportion to the spatial frequency. Specifically, the only stimulus transformation across species was the depth from the monitor. This global scaling preserves the number of cycles present with the Gaussian mask. These distances were chosen in order to render the stimulus with a spatial frequency that is comparably sensitive for each species’ behaviorally measured contrast sensitivity (Keller et al., 2000). Third, both humans and rats viewed stimuli in which the spatial context surrounding the target varied randomly on each trial. As a consequence, a subject could not rely on a flanking stimulus to appear at a particular position or to have a particular orientation. Nor would the subject know that the next stimulus was going to be a particular orientation. This experimental paradigm should prevent a subject from ignoring a particular orientation, which might have been a good strategy if there had been a block of trials in which the target orientation was constant and differed from the flanker orientation.

#### Training

Rats were trained to perform the task by progressing through a sequence of five shaping steps, as previously described (Meier et al., 2011). To summarize the training steps, rats first learned to detect a large grating, which was then decreased in contrast, increased in spatial frequency, reduced in spatial extent, and was finally embedded in a spatial context with flankers of increasing contrast. This training process took rats multiple weeks to complete, with a 2-h session each day. Most of the training was spent on the last two stages. Humans began immediately on the final task. They learned to perform it over hundreds of trials, all in a single session. For humans, testing and training occurred on the same day, in a single 2-h session. Qualitatively, both rats and humans learned the task through trial and error. Quantitatively, rats observed many more trials before attaining adequate performance.

#### Display

Stimuli were presented on a CRT monitor (100 Hz, 1024 × 768 pixels). When humans performed the exact same task as the rats, they were close to 100% correct (preliminary study, data not shown). To increase the difficulty of the task for humans, the contrast of the target was reduced, *T*_c_ = [0.0625]. The contrast of the flankers was kept the same as was used for the rats, *F*_c_ = [1.0]. Additionally, the stimulus duration was reduced to 100 ms. During training, as well as the first experiment, rats were allowed to view the stimulus indefinitely.

Stimuli were presented on a monitor 10 cm from the rat’s eyes. It is possible that rats’ acuity or sensitivity is higher at other viewing distances. Optimal viewing distances for Long Evans (hooded) rats have been reported to be between 20 and 30 cm (Wiesenfeld and Branchek, 1976), and many behavioral studies present stimuli at depths within this range (Lashley, 1930; Birch and Jacobs, 1979; Dean, 1981; Alemi-Neissi et al., 2013). Yet other studies report that, compared to 30 cm, detection sensitivity did not consistently decrease at proximal depths like 12 cm (Dean, 1981) or 15 cm (Birch and Jacobs, 1979). Successful visual experiments have been performed on touch screens with display surfaces as close as 7 cm (Keller et al., 2000) or 2 cm (Bussey et al., 2008). We chose 10 cm as a viewing distance for experimental convenience; the compact arrangement of training chamber and monitor allowed a rack of nine simultaneously operating rigs to occupy a small footprint of floorspace. After selecting a distance, we chose a contrast and spatial frequency that yielded detection above perceptual threshold, favoring high contrast and a moderately high spatial frequency.

## EXPERIMENT 2

A second experiment was performed for two of the rats. To adequately sample many combinations of target contrast and flanker contrast, all spatial parameters were held constant. Thus, if flankers were present, they were collinear (**Figure 3**). In this experiment, there were twenty possible stimulus conditions: four target contrasts (*T*_c_ = [0.25, 0.5, 0.75, 1]), and five flanker contrasts ([*T*_f_ = 0, 0.25, 0.5, 0.75, 1]). On each block, the flanker contrast was constant, and the target, if present, was also constant. On half of the trials, the target was not present (*T*_c_ = 0). Conditions were randomly assigned to a block of 100 trails. One subject performed an average of 485 trials per day, resulting in 21 blocks per stimulus condition; the other subject performed an average of 585 trials per day resulting in 27 blocks per stimulus condition. The stimulus was present for 200 ms on each trial. In all other respects the methods were the same as for Experiment 1. Data were collected in 96 sessions over 101 days.

### DATA COLLECTION

Rat behavioral data was collected from seven male Long Evans rats (Harlan Laboratories) and four university student volunteers. Experiments were conducted under the supervision and with the approval of either the Human Research Protections Program or the Institutional Animal Care and Use Committee at the University of California San Diego.

The rodent data is from the same trained rats and the same experimental protocol as previously reported (Experiment 1: Meier et al., 2011; Experiment 2: Meier and Reinagel, 2011) but the data have been analyzed differently. Specifically, we report performance with flankers in relation to each subject’s detection performance of the target alone. Additionally, we have grouped the performance estimate of the three types of non-collinear stimuli, because they were not significantly different from each other in our analysis. The human data were collected to approximate the same task as the one performed by the rats, and was analyzed the same way. One human subject was excluded from analysis because they never learned to perform the task above chance.

### ANALYSIS

Behavioral performance is reported as both the fraction of correct trials and *d*’. The former provides an intuitive sense of the raw data; the latter is a metric of signal detection theory that aims to separate a subject’s sensitivity to the target from errors due to their bias to choose a particular response.

Confidence intervals in **Figure 2C** were generated using a permutation test that would reject the hypothesis that a subject’s sensitivity to two stimulus categories was equal. Each trial has a stimulus identity (e.g., collinear or non-collinear) and the subject’s response (e.g., reporting that the target was present or absent). The subject’s response was randomly permuted within all trails with a target, and again within all trials without a target, destroying the relationship between the stimulus identity and the response. *d*’ was computed for each of the two stimulus categories (collinear and non-collinear) and the difference between the two was computed. The permutation and the analysis was repeated 10,000 times, resulting in a distribution of differences that would be expected if the sensitivity was not different. The top and bottom 250 samples were removed, providing an estimate of the boundary that would contain the observed measure 95% of the time, if the null hypothesis were true.

## RESULTS

Both humans and rats performed the same task to detect a faint target. During each trial of the task the configuration of the flankers was randomly varied (**Figure 1**). The many possible stimulus patterns were organized into three groups for analysis: trials without any flanking stimuli (“no flanker”), trials with two collinear flankers (“collinear”), and trials with two flankers present, neither of which was collinear to the target (“non-collinear”). These three non-overlapping categories fully contained all stimuli presented to the subjects. Both humans and rats learned to perform the task above chance levels. Humans learned the task and were tested in the course of a single session; the average performance of a single human ranged between 60% and 80% correct. Rats learned over the course of many weeks; the average performance for rats during the testing phase was between 60% and 70% correct. The absolute performance of each subject was not of particular interest, beyond confirming that it was it belonged to a range that could potentially reveal improvements or impairments.

To isolate the impact of collinear flankers, we compare a subject’s detection performance between the stimulus types (**Figure 2**). Each human subject performed better on trials with collinear stimuli than on trials with non-collinear stimuli (significant in 2 of 4). This is consistent with reports that humans can detect fainter contrasts when flanking stimuli are collinear to the target (Polat and Sagi, 2007). On the other hand, each rat performed worse on collinear than non-collinear stimuli (significant in 6 of 7), as previously reported in rats. Notably, the rats’ behavior reveals that their visual system is specifically influenced by collinear flankers, above and beyond the influence to non-collinear flanking stimuli. However, the additional impact of collinear stimuli is to impair, rather than improve their performance. The absolute effect of flankers also differed between species: each human subject performed better on trials with flankers (collinear or not) than on trials without flankers; each rat performed worse when flankers were present.

Could the difference in contrast of the target alone explain the differences observed between rats and humans? Previous findings about human performance suggest that the impact of flankers on target detection at high contrasts may be different than on threshold target detection at low contrasts (Williams and Hess, 1998). Moreover, studies in human psychophysics as well as mammalian neurophysiology (Seriès et al., 2003) suggest that in some circumstances, the relative contrast of flanker to target could switch the influence of flankers from facilitative to suppressive. Because the humans were better at performing the detection task in a pilot study, the contrast of the target had been set to a lower value for humans in the first experiment, to achieve detection performance near threshold. Might the observed collinear impairment disappear, or even invert (Polat et al., 1998), if rats view collinear flankers that are substantially higher contrast than the target? Importantly, it was not known if the contrast of the target alone is the parameter that matters, or if the relative contrast of the target to the flanker matters more.

To address these questions, in a second experiment, two rats were tested with many combinations of target contrast and flanker contrast. This experiment includes a condition where that flanker contrast is four times as large as the target contrast (**Figure 3A**), as well as a condition where the flanker contrast is one quarter the strength of the flanker contrast (**Figure 3B**), as well as many steps in between. We want to know if there is a contrast regime where rats will perform better in the presence of collinear flankers compared to no flankers (**Figure 2B**, the combined length of both arrows). Specifically, will the sign of the effect ever invert for rats, such that collinear flankers improve detection performance, as they do for humans? We find the answer is no.

In none of the tested cases do collinear flankers improve rats’ detection (**Figure 4**). More specifically, for each target contrast, “no flank” performance was always better than a “collinear” stimulus with a matched target contrast. As the target contrast is increased, the “no flank” condition improved more than the “collinear condition,” increasing the difference in performance between the conditions (**Figures 4A,D**). In other words, given an increment of target contrast, rats were less sensitive to the additional signal when the collinear flankers were present. As the flanker contrast increased, the impairment caused by flankers increased (**Figures 4B,E**). This rules out the hypothesis that higher contrast flankers might improve rats’ detection, either by creating a sub-threshold pedestal for the low contrast target (Chen and Tyler, 2002) or by providing a consistent salient visual anchor for spatial attention (Petrov et al., 2006). To restate, all tested conditions (**Figures 4C,F**) produced a collinear impairment in both rats.

## DISCUSSION

The goal of this study was to directly compare collinear processing between humans and rats, and to synthesize findings in both species for a better understanding of canonical cortical computations.

We tested humans and rats on the same detection task. In both species, flankers with collinear spatial patterns had the strongest effect on performance. However, the nature of the collinear effect is strikingly different between the species: collinear features helped humans perform the task, but they impaired rats. This makes it unlikely that the previously reported difference was due to differences in task design or contrast regime. Instead, it appears that some aspects of visual processing, specifically regarding correlations of spatially adjacent features, differs between rodent and human vision.

This is the first report of human performance on a detection task with oriented flankers where the experimental design randomized target and flanker orientations on every trial. This design prevents subjects from using the spatial pattern of the previous trial to attend to features that would make the detection task easier. Our results provide evidence against the model that feature-based attention underlies collinear facilitation in humans.

### POSSIBLE EXPLANATIONS OF OBSERVED SPECIES DIFFERENCE

Some of the behavioral consequences of flanking stimuli are likely mediated by lateral interactions between the cortical layers that represent both the target features and the flanking features. However, we cannot rule out that features may interact via the computations of higher order visual features, or even via the subject’s decision process.

Our prior was that the detection of collinear contours would be a fundamental visual computation conserved across mammals. The different effect of collinear stimuli in rats and humans therefore came as a surprise; we do not have an explanation for it. Below we consider five hypotheses that could explain our observations: between humans and rats there may be (1) differences in task understanding, (2) differences in recent visual experience, (3) differences in the statistics of natural retinal images (4) difference in neural resources and thus over-completeness of pattern representations (5) differences in attention.

#### (1) A difference in how humans and rats understand the task

In this study, both rats and humans inferred the task goal by trial and error, without explicit instructions. All human subjects were given an exit survey. Of the four included in this study, three subjects were able to articulate the stimulus properties they used to answer correctly, such as attending to the region between the two flankers. One subject was not able to articulate which visual properties influenced their judgments. Strikingly, this subject was above chance, yet did not seem to understand what the task was. Indeed, this subject was not even aware of performing the task better at the beginning or the end of the session. Given that this human subject did not understand the task, and hypothesizing that the rats did not understand the task, is the subject’s performance consistent with the rats? The answer is no: all humans tested (who were above chance) had the same collinear facilitation, even the one that did not seem to understand the task. Based on this anecdotal evidence, we do not favor differences in task understanding as an explanation of the species difference. Of course, despite our efforts to match the learning procedures, there could still be differences between humans and rats about how they understand the task.

#### (2) A difference in visual experience in the training phase

Since human training was very short (about 15 min, and a few hundred stimuli) the preceding stimuli might have had a different effect than for the rats, who viewed more stimuli (over months, tens of thousands of trials). Additionally, humans only viewed the final task, whereas rats were shaped to perform the task through a series of shaping steps. One of these steps included a large target. Therefore it is possible that the rats learned to use information that was collected from the region that the flankers were going to occupy in later testing phases, and failed to unlearn that these regions were in fact foils during the testing phase. However, we note that the number of trials with large targets was small (hundreds of trials, months ago) compared with the majority of trials in which the target and the surround were independent. Therefore we think it is unlikely that this explains the full reversal of the collinear effect across species.

#### (3) A difference in anatomy and visual experience across evolution

Natural scenes are self-similar (Tolhurst et al., 1992; Ruderman and Bialek, 1994; van der Schaaf and van Hateren, 1996). Thus, the statistics of collinear line elements should be similar across scales spanned by rat vision and human vision. However, humans and rats see the world from a different point of view. Rats’ eyes are closer to the floor, and they rarely shift their gaze vertically (Chelazzi et al., 1989). This latter fact may be particularly important because it seems that the rodent retina has adapted to different evolutionary constraints for the stimuli in different spatial locations. For example, the upper visual hemifield contains a different distribution of cones than the lower hemifield (Ortín-Martínez et al., 2010), possibly due to representing different features in the land and sky. In this study, rats viewed stimuli that were in the upper visual field. Rats have lower visual acuity than humans (Keller et al., 2000), a smaller fraction of cones than humans (Kimble and Williams, 2000). Taken together, these differences in the early visual system suggest that at an evolutionary time scale, the statistics of visual input was different for rats, and that the visual system optimized differently to represent them. It could be argued that spatial vision in rats is different in their upper and lower hemifields. The lower hemifield may be used to detect and identify the spatial patterns of nearby visual objects, and the upper visual field may be more relevant for more distant cues such as landmarks or swooping predators. The limited optical range of the rodent eye may render the retinal image blurry for distant objects. In summary, humans may have more evolutionary experience bringing structured objects into focus, regardless of their depth, and thus more opportunity and selective pressure to evolve mechanisms exploiting the relative correlations of local image features. This could explain why rats might lack collinear facilitation, at least in the upper visual field. But it fails to account for collinear-specific impairment.

#### (4) Flankers impair performance by crowding, but primates have mechanisms to combat crowding

One possible explanation is that all flanking stimuli cause a universal impairment in target detection, but that some organisms have attentional and/or perceptual resources that capitalize on collinear edges and overpower the deficit of crowding. If there are fewer cortical neurons to represent each square degree of a visual scene (as in a rodent or the primate para-fovea), the impact of crowding may be stronger, and the deficit observed in from the presence of flankers will be greater. Indeed, crowding from a distant flanker is stronger in the para-fovea than in the fovea of humans (Levi, 2008), and rats, compared to humans, display greater detection deficits in the presence of flankers. Crowding could account for the pattern of deficits observed in the range of contrasts conditions tested in rats (**Figure 4**; Levi and Carney, 2011; Meier and Reinagel, 2011). Crowding could explain why any flanker might impair target detection, and why collinear features, or more proximal features, or higher contrast features would impair more. However, crowding will not explain the benefits in target detection that are conferred to humans when flanking stimuli are present. To explain the improvement, one would have to posit an additional resource, unique to primates and absent in rodents.

#### (5) Collinear facilitation requires selective visual attention, which is more developed in primates

Collinear facilitation could be a hallmark of deployed attention. Previous studies suggest that collinear facilitation in humans depends on the allocation of attention (Freeman et al., 2001). Many anatomical structures in the deployment of spatial visual attention overlap with the neural resources involved in the guidance of eye movements – notably the superior colliculus and frontal eye fields. Rats do not have a fovea, and lack the rich saccadic eye movements found in primates. Therefore rats may lack specializations of the spatial visual attention system that primates have evolved in association with saccadic foveation. A difference in attentional mechanisms could explain the cross-species differences in the detection task observed in this paper.

The five explanations considered above are speculative, and not mutually exclusive. We suspect the difference is due to a combination of the latter three: differences in the statistics of the natural retinal images, the representational capacity of neurons in visual cortex, and the attention mechanisms of an organism.

In closing, the neural mechanisms of collinear interactions remain unknown in either species. We presented strong evidence that processing of collinear features is different between rats and humans. Elucidating the circuit mechanisms in either species would be of great value, and the best model would be one that could account for the differences between the species.

## Conflict of Interest Statement

The authors declare that the research was conducted in the absence of any commercial or financial relationships that could be construed as a potential conflict of interest.
